# Editorial: Recent Advances in Bladder Cancer Diagnosis and Treatment

**DOI:** 10.3389/fsurg.2022.890172

**Published:** 2022-04-12

**Authors:** Jeremy Yuen-Chun Teoh, Daniele Castellani

**Affiliations:** ^1^S.H. Ho Urology Centre, Department of Surgery, The Chinese University of Hong Kong, Hong Kong, Hong Kong SAR, China; ^2^Faculty of Medicine, Urology School, Università Politecnica delle Marche, Ancona, Italy

**Keywords:** bladder cancer, urothelial carcinoma, urinary marker, lymphadenectomy, chemohyperthermia, BCG - bacille calmette-guérin vaccine, transurethral resection (TUR) of bladder

According to GLOBOCAN data, bladder cancer is the 10^th^ most common cancer worldwide, and there were 573,278 estimated new cases and 212,536 death worldwide in 2020 (1). Men are affected consistently higher than women with a male: female ratio ranging from 2:1 to 6:1 (2). The incidence of bladder cancer increases steeply after the age of 50 years, particularly due to exposures to risk factors such as cigarette smoking and occupational exposures in developed countries, and Schistosomiasis infection in Africa and the Middle East (3). Two third of bladder cancer cases are non-muscle-invasive at diagnosis and tend to recur during patient life.

The world population is expected to increase from the current 7.6 billion to 8.5 billion people in 2030, and this demographic change will have undoubtedly a huge influence on bladder cancer occurrence, prevalence, and mortality with a growing burden on clinical care (4). Despite various types of surgical and systemic treatments, the oncological outcomes of bladder cancer are still unsatisfactory.

Recently, there have been a lot of advances in the diagnosis and treatment of bladder cancer. Although cystoscopy is the gold standard in the primary diagnosis and surveillance of bladder cancer, urinary markers and enhanced imaging are likely to play an important role in the future. In addition, newer surgical approaches have been adopted in the management of bladder cancer. The main aim of this Research Topic was to cover promising, recent, and future research trends in the field of Bladder Cancer, with straightforward key messages for clinicians interested in this field. This special Research Topic was launched in January 2021 and closed in September 2021. Within 8 months, 15 manuscripts were submitted, of which 9 were accepted. There were 6 reviews and 3 original articles. By February 2022, this special Research Topic reached 14,374 views. The subjects covered were urine biomarkers (Chai et al.; Sugeeta et al.), en bloc resection of bladder tumor (Fankhauser et al.; Liu et al.), the adequacy of pelvic lymphadenectomy during radical cystectomy (Jena et al.), the role of two T1 sub staging systems on recurrence and progression (Asimakopoulos et al.), the role of macroscopic image enhancement in the diagnosis of non-muscle-invasive bladder cancer (Mulawkar et al.), the role of pathologists in handling and reporting bladder cancer samples (Mazzucchelli et al.), and intravesical chemohyperthermia vs. bacillus Calmette-Guerin instillation for intermediate- and high-risk non-muscle-invasive bladder (Zhao et al.).

Just to highlight some key messages from the articles. Chai et al. compared CxBladder, a new mRNA biomarker, with conventional urine cytology and found that the former had a high negative predictive value and sensitivity that accurately predicted suspicious cystoscopy findings. Sugeeta et al. reviewed current biomarkers and summarized the findings of each marker in clinical practice. Mulawkar et al. reviewed the role of the macroscopic image enhancement in the diagnosis of non-muscle-invasive bladder cancer which demonstrated a great utility in improving detection and short-term cancer control but no utility in delaying progression, or in long-term cancer control. Fankhauser et al. reviewed the current role of en bloc resection of bladder tumors, showing the improvement in clinical outcomes of the en bloc procedures. This result was confirmed by Liu et al. who showed that Thulium laser en bloc resection was safer than conventional transurethral resection with fewer perioperative complications. Asimakopoulos et al. analyzed the importance of T1 sub staging in non-muscle-invasive bladder cancer and showed that extensive invasion of the lamina propria was significantly associated with recurrence-free survival and progression-free survival. Jena et al. reviewed and analyzed the different anatomic templates of pelvic lymph node dissection during radical cystectomy, based on levels of pelvic lymph nodes. Mazzucchelli et al. provided important information on handling and reporting of the bladder cancer samples to improve the close collaboration between pathologists and urologists. Finally, Zhao et al. showed in a systematic review that intravesical chemohyperthermia had equivalent oncological outcomes and a similar safety profile when compared to BCG maintenance therapy for patients with intermediate- and high-risk non-muscle-invasive bladder cancer.

This Research Topic represents the hard work of all the authors and reviewers, who have contributed significantly to make this possible. We hope that you will enjoy reading this special Research Topic as we did in handling all the papers.

## Author Contributions

All authors listed have made a substantial, direct, and intellectual contribution to the work and approved it for publication.

## Conflict of Interest

The authors declare that the research was conducted in the absence of any commercial or financial relationships that could be construed as a potential conflict of interest.

## Publisher's Note

All claims expressed in this article are solely those of the authors and do not necessarily represent those of their affiliated organizations, or those of the publisher, the editors and the reviewers. Any product that may be evaluated in this article, or claim that may be made by its manufacturer, is not guaranteed or endorsed by the publisher.
